# ﻿The systematic status of *Afropisasanctaehelenae* (Chace, 1966) (Decapoda, Brachyura, Majoidea, Epialtidae): morphological and molecular evidence

**DOI:** 10.3897/zookeys.1243.145270

**Published:** 2025-06-27

**Authors:** Isabel Muñoz, Paul F. Clark, Jose A. Cuesta

**Affiliations:** 1 Centro Oceanográfico de Cádiz, Instituto Español de Oceanografía (IEO-CSIC), Puerto Pesquero, Muelle de Levante, 11006, Cádiz, Spain Centro Oceanográfico de Cádiz, Instituto Español de Oceanografía (IEO–CSIC) Cádiz Spain; 2 Department of Life Sciences, The Natural History Museum, Cromwell Road, London, SW7 5BD, UK Department of Life Sciences, The Natural History London United Kingdom; 3 Instituto de Ciencias Marinas de Andalucía (ICMAN-CSIC), Avda. República Saharaui, 2, 11519, Puerto Real, Cádiz, Spain Instituto de Ciencias Marinas de Andalucía (ICMAN–CSIC) Cádiz Spain

**Keywords:** 16S, Cox1, crab, Crustacea, DNA barcoding, Saint Helena Island

## Abstract

*Afropisasanctaehelenae* (Chace, 1966) was originally described from three specimens collected from Saint Helena Island, South Atlantic, and assigned to *Pisa* Leach, 1814. It is a poorly known species, and until now, no new records have been reported. Recently, this species was transferred to a new genus, *Afropisa* Muñoz, García-Raso, González, Lopes, dos Santos & Cuesta, 2023, based exclusively on morphology. Nearly 60 years on from the original description, another eight specimens of *A.sanctaehelenae* have been made available for further study and sequencing. Mitochondrial 16S rRNA and cytochrome c oxidase subunit I sequences confirm the assignment of this Chace species to *Afropisa*. Photographs of the male holotype and female paratype confirmed the presence of additional characters; consequently, the present work provides a redescription of this species.

## ﻿Introduction

The superfamily Majoidea Samouelle, 1819, comprises seven families, including Epialtidae MacLeay, 1838. The epialtid genus, *Afropisa* Muñoz, García-Raso, González, Lopes, dos Santos & Cuesta, 2023, was recently established comprising three species: *A.calva* (Forest & Guinot, 1966), *A.carinimana* (Miers, 1879), and *A.sanctaehelenae* (Chace, 1966) (Muñoz et al. 2023). All are of a relatively small sized epialtids distributed along West Africa, with *A.carinimana* being reported as common in western Mediterranean waters (Chace 1966; Forest and Guinot 1966; Zariquiey Alvarez 1968; d’Udekem d’Acoz 1999; García Raso et al. 2006; Marco-Herrero et al. 2015; González 2018; Muñoz et al. 2023). *Afropisa* was established by Muñoz et al. (2023) for these three species because they shared several morphological characteristics, which differentiate them from species assigned to *Pisa* Leach, 1814. Although Muñoz et al. (2023) only obtained 16S and Cox1 sequences for *A.carinimana*, their study distinguished it from the other *Pisa* species and further demonstrated a relationship with *Micropisa* Stimpson, 1857. Therefore, based exclusively on morphological characters, Muñoz et al. (2023) assigned *A.calva* and *A.sanctaehelenae* to *Afropisa*. *Afropisasanctaehelenae* types deposited in the United States National Museum, Smithsonian Institution, Washington DC, USA, were not available at that time for study. Furthermore, a positive PCR could not be obtained from *A.calva* specimens loaned by the Naturalis Biodiversity Center, The Netherlands.

**Figure 1.**
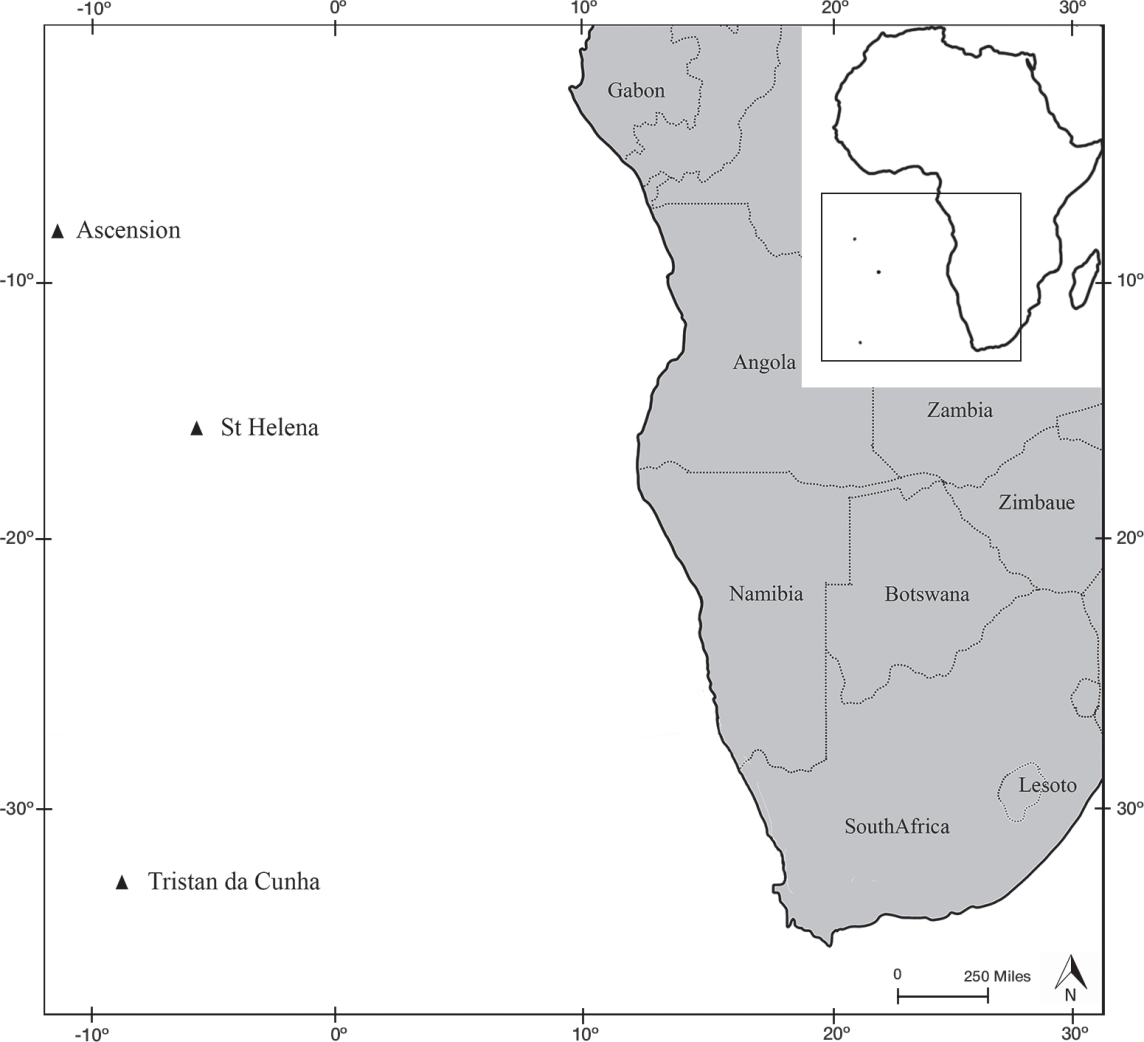
Map of Southwest Africa, including the islands of Ascension, Tristan da Cunha, and Saint Helena (based on Google maps).

Until the recent publication of Brown (2014), only a few studies involved the brachyuran crabs of the Tristan da Cunha Archipelago comprising the islands of Saint Helena, Ascension, and Tristan da Cunha (Studer 1883; Chace 1966; Manning and Chace 1990; den Hartog and Türkay 1991). Saint Helena and Ascension are isolated volcanic islands (Manning and Chace 1990), located in the South Atlantic and separated from each other by more than 1300 km (Fig. 1). In an inventory of the crustaceans collected during the cruiser of S.M.S. *Gazelle* on the west coast of Africa, Ascension, and the Cape of Good Hope, Studer (1883) only cited the epialtids *Pisaarmata* (Latreille, 1803), as *P.gibbsii* Leach, 1816, and *Micropisaovata* Stimpson, 1857. Chace (1966), however, listed 23 species of decapod crustaceans from Saint Helena, including the description of two new species *Acanthonyxsanctaehelenae* Chace, 1966, and *Pisasanctaehelenae* Chace, 1966. Subsequently, Manning and Chace (1990), in their catalogue of decapod and stomatopod crustaceans from Ascension Island, reported *A.sanctaehelenae*, from Ascension and Saint Helena, *Apiomithraxviolaceus* (A. Milne Edwards, 1867) only from Ascension Island, and *P.sanctaehelenae* only from Saint Helena.

The present study aims to barcode fresh material of *Afropisasanctaehelenae* from Saint Helena and provide additional characters to clarify the current systematic assignment of this species.

## ﻿Material and methods

### ﻿Morphological study

Eight *A.sanctaehelenae* specimens collected from Saint Helena Island are morphologically studied in detail. In addition, photographs provided by the National Museum of Natural History, Smithsonian Institution, of the male holotype and female paratype of *A.sanctaehelenae* were carefully examined.

The terminology used in the descriptions and comparisons follows Davie et al. (2015). Measurements and abbreviations used in the text and figures are: **cl** = carapace length, measured along the dorsal midline and includes the rostral spines; **cw** = carapace width, the greatest width across the branchial regions, excluding lateral spines; **pcl** = postrostral carapace length, measured along the dorsal midline from the base of the sinus between the rostral spines and the posterior margin of the carapace.

Other abbreviations used: ♀ = female; ♂ = male; **CCDB** = Crustacean Collection of the Department of Biology of FFCLRP, University of São Paulo, Ribeirão Preto, Brazil; **ICMZ** = Zariquiey Marine Crustacean Legacy Collection (Santos-Bethencourt et al. 2025); **IEOCD** = Marine Crustacean Collection of the Cádiz Oceanographic Center (Muñoz and Serrano 2024); **MMF** = Natural History Museum of Funchal Collection, Madeira; **NHMUK** = Natural History Museum of United Kingdom collection (Natural History Museum 2025); **ovig.** = ovigerous; **P1**–**P5** = first to fifth pereiopods; **UB** = University of Barcelona; **USNM** = National Museum of Natural History, Washington DC (formerly the United States National Museum).

### ﻿Molecular analysis

Total genomic DNA of two out of the eight *A.sanctaehelenae* specimens studied herein was extracted from muscle tissue from one pereiopod, following a modified Chelex 10% protocol by Estoup et al. (1996). Target mitochondrial DNA from the 16S rRNA (16S) and cytochrome c oxidase subunit I (Cox1) genes was amplified with polymerase chain reaction (PCR) using the following cycling conditions: 2 min at 95 °C, and 35 cycles of 30 s at 95 °C, 30 s at 44–54 °C (depending on primer combination), and 30 s (16S) or 45 s (Cox1) at 72 °C, and a final 5 min at 72 °C. Primers 1472 (5′–AGA TAG AAA CCA ACC TGG–3′) (Crandall and Fitzpatrick 1996), 16L2 (5′–TGC CTG TTT ATC AAA AAC AT–3′) (Schubart et al. 2002), and 16L12 (5′–TGA CCG TGC AAA GGT AGG ATA A–3′) (Schubart et al. 1998) were used to amplify a minimum of 450 bp and a maximum of 540 bp of 16S, while primers COH6 (5′–TAD ACT TCD GGR TGD CCA AAR AAY CA–3′) and COL6b (5′–ACA AAT CAT AAA GAT ATY GG–3′) (Schubart and Huber 2006), and LCO1490 (5′–GGT CAA CAA ATC ATA AAG ATA TTG–3’) and HCO2198 (5’–TAA ACT TCA GGG TGA CCA AAA AAT CA–3′) (Folmer et al. 1994) allowed amplification of a maximum of 658 bp of Cox1. PCR products were sent to Stab Vida company to be purified and then bidirectionally sequenced.

Sequences were edited using the software CHROMAS LITE 2.6.4 (Technelysium Pty Ltd, 2017) and aligned with BIOEDIT Sequence Alignment Editor 7.2.6.1 (Hall 1999). The final DNA sequences obtained were compared with sequences retrieved from the GenBank database. New sequences have been deposited in GenBank under the accession numbers PQ788831 to PQ788832 (16S) and PQ788866 to PQ788867 (Cox1).

Phylogenetic and molecular evolutionary analyses were conducted using MEGA version X (Kumar et al. 2018) on the new sequences obtained for *A.sanctaehelenae* and other sequences of *Pisa*, *Afropisa*, and *Micropisa* species downloaded from the GenBank. The best-fitting nucleotide substitution model for 16S and Cox1 was obtained with the tools implemented in MEGA X, using the corrected Akaike information criterion. The analyses for 16S and Cox1 were carried out separately because of the composition of different species/specimens for each gene. The phylogenetic reconstruction analyses for 16S and Cox1 sequences databases were performed with maximum-likelihood (ML) analysis using MEGA X. Topological robustness was tested using 2000 nonparametric bootstrap replicates. In the analysis of both genes, *Herbstiacondyliata* (Fabricius, 1787), *Apiomithraxviolaceus*, *Apiomithraxbocagei* (Ozorio, 1887), *Samadiniagalathea* (Griffin & Tranter, 1986), and *Samadiniapulchra* (Miers in Tizard, Moseley, Buchanan & Murray, 1885) were used as the outgroup.

## ﻿Results

### ﻿Morphological study

Systematic account

**Superfamily Majoidea Samouelle, 1819**

**Family Epialtidae MacLeay, 1838**

**Genus *Afropisa* Muñoz, García-Raso, González, Lopes, dos Santos & Cuesta, 2023**

#### ﻿ Afropisa sanctaehelenae

Taxon classificationAnimaliaDecapodaEpialtidae

(Chace, 1966)

284276FB-E3AA-5009-91E7-10800F85CDB9

**Figure 2.**
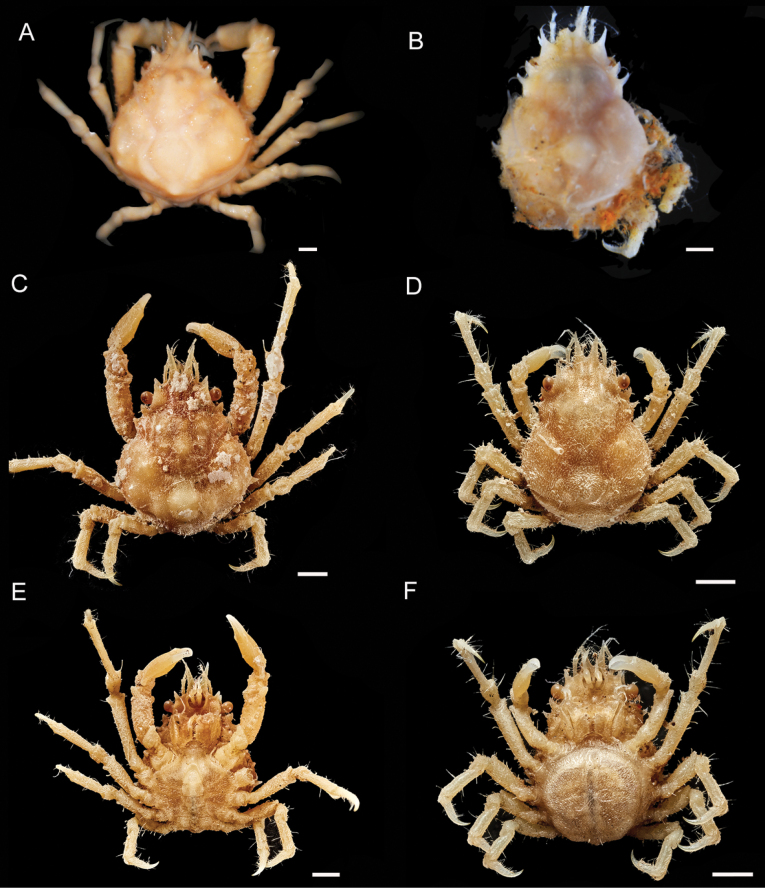
*Afropisasanctaehelenae* (Chace, 1966) **A** ♂ holotype (USNM 112459), dorsal view **B**ovig. ♀ paratype (USNM 112460), dorsal view **C** ♂ (NHMUK 2024.240), dorsal view **D**ovig. ♀ (NHMUK 2024.239), dorsal view **E** ♂ (NHMUK 2024.240), ventral view **F**ovig. ♀ (NHMUK 2024.239), ventral view. Scale bars: 1 mm. (**A, B** photos by Karen Reed and Lisa Comer, Smithsonian Institution **C–F** photos by Jonathan Jackson, NHM Publishing and Image Resources).

**Figure 3.**
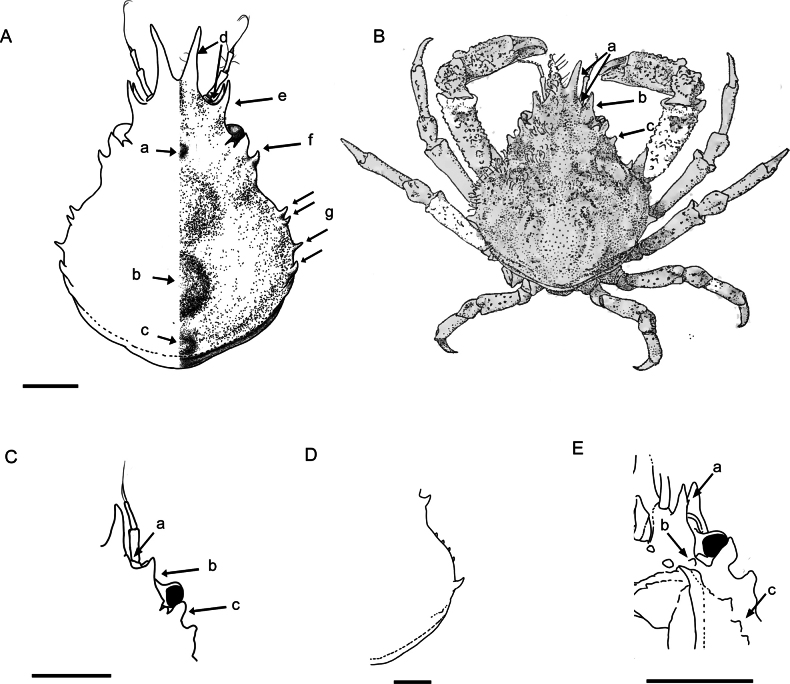
*Afropisasanctaehelenae* (Chace, 1966) **A** ♂ (NHMUK 2024.232), dorsal view of carapace (abbreviations: a = mesogastric tubercle; b = cardiac region; c = intestinal region prominence; d = rostral spine and supraorbital margin angle; e = supraorbital margin projection; f = hepatic spine; g = lateral spines) **B** ♂ holotype (USNM 112459), dorsal view (abbreviations: a = rostral spine and supraorbital margin angle; b = supraorbital margin projection; c = hepatic spine) **C**ovig. ♀ (NHMUK 2024.233), anterior and orbital region (abbreviations: a = rostral spine and supraorbital margin angle; b = supraorbital margin projection; c = hepatic spine) **D**ovig. ♀ (NHMUK 2024.238), lateral spines **E** ♂ (NHMUK 2024.232), suborbital region, ventral view, basal antennal article (abbreviations: a = distal minute spine on spine of basal antennal article; b = tooth in posterolateral angle of basal antennal article; c = row of acute pterygostomian teeth). Scale bars: 1 mm (**B** modified from Chace 1966).

**Figure 4.**
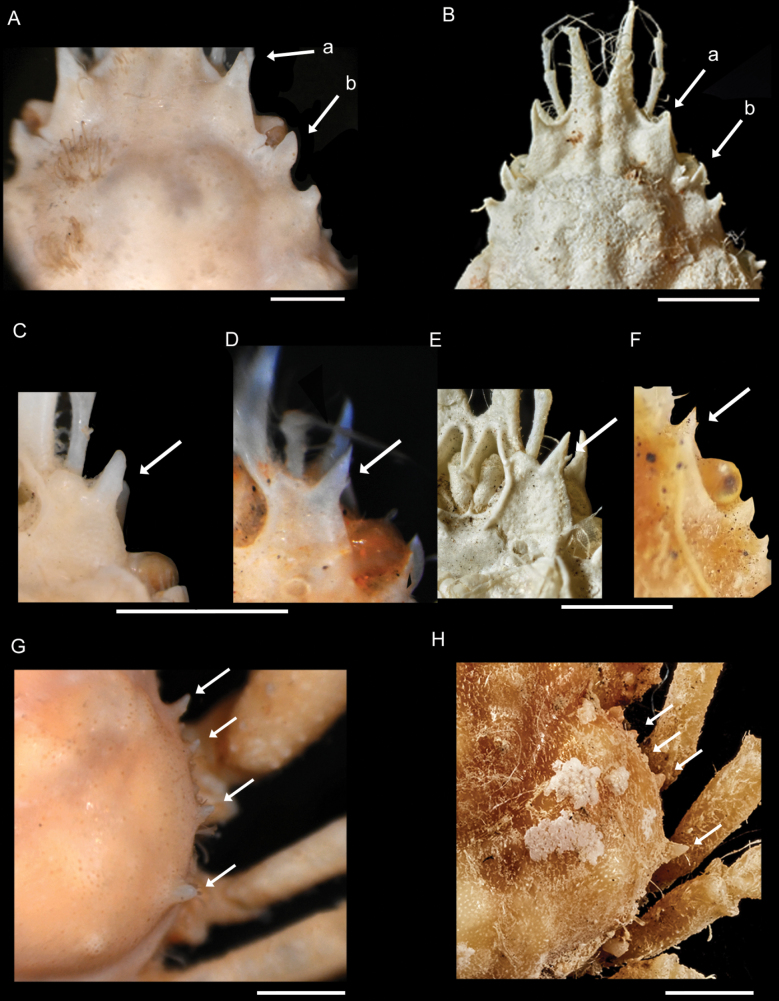
*Afropisasanctaehelenae* (Chace, 1966) **A** ♂ holotype (USNM 112459) **B** ♂ (NHMUK 2024.232) **C** ♂ holotype (USNM 112459) **D**ovig. ♀ paratype (USNM 112460) **E** ♂ (NHMUK 2024.232) **F** ♂ (NHMUK 2024.236) **G** ♂ holotype (USNM 112459) **H** ♂ (NHMUK 2024.236) **A, B** anterior and orbital region (abbreviations: a = long spine in supraorbital margin; b = flat plate-shaped postocular process) **C–F** suborbital region, ventral view, basal antennal article (arrow indicates small distal minute spine) **G, H** lateral spines. Scale bars: 1 mm (**A, C, D, G** photos by Karen Reed and Lisa Comer, Smithsonian Institution **B, E, H** photos by Jonathan Jackson, NHM Publishing and Image Resources).

Table 1 , Figs 2 , 3 , 4

Pisa sanctaehelenae Chace, 1966: 651–654, fig. 14a–h; Brown 2014: 48, 2 colour plates.
Afropisa sanctaehelenae Muñoz et al., 2023: 12, fig. 5H.

##### Type material

**(examined from photographs). *Holotype*.** Saint Helena Island • 1 ♂ (11.3 mm pcl, 13.2 mm cl, 9.7 mm cw); off Rupert’s Bay; 11 Feb. 1963; in a buoy between 0–2 m; USNM 112459.

***Paratype*.** Saint Helena Island • 1 ovig. ♀ (5.8 mm pcl, 7.0 mm cl, 4.3 mm cw); off Rupert’s Bay; 11 Feb. 1963; in a cable of a buoy between 0–2 m; USNM 112460.

##### Other material examined.

Saint Helena Island • 1 ♂ (5.3 mm pcl, 6.5 mm cl, 4.5 mm cw); locality not specified; 29 Jun. 1983; C. Chass and A. Edwards leg.; in algal mat at water edge; NHMUK 2024.232 • 1 ovig. ♀ (4.3 mm pcl, 5.2 mm cl, 3.6 mm cw); data on locality, date, and collector not specified; GenBank: PQ788831 (16S), PQ788866 (Cox1); NHMUK 2024.233 • 2 ♂♂ (3.3 and 6.3 mm pcl, 4.1 and 7.3 mm cl, 2.5 and 5.5 mm cw), 4 ovig. ♀♀ (3.9, 4.1, 4.4 and 4.6 mm pcl, 4.2, 4.8, 5.3 and 5.3 mm cl, 2.9, 3.4, 4.0 and 4.1 mm cw); at Rupert’s Bay, near the jetty, 5.2 m depth from zoanthid colony; 20 Jan. 2014; Jude Brown leg.; GenBank: PQ788832 (16S), PQ788867 (Cox1) [for NHMUK 2024.235]; NHMUK 2024.235–240.

##### Redescription

**(based on the description of Chace 1966).** Carapace subpyriform, longer than wide, regions well delimited (Fig. 2). Carapace (excluding rostral and lateral spines) 1/6 to 1/3 longer than broad. Postrostral carapace length (pcl)/carapace width (cw) = 1.10–1.34. Mesogastric region bulging and prominent, with 1 small anterior tubercle (Fig. 3Aa); cardiac region highly convex or swollen, surrounded by deep furrow; both regions without spines (Fig. 3Ab). Intestinal region with median prominence (Fig. 3Ac). Branchial regions bearing lateral boss, obscure longitudinal sulcus behind cervical groove and three smaller prominences in oblique row across mesobranchial region; posterior most forming blunt tooth on posterolateral margin. Surface pubescence sparse; groups of hooked setae on rostral spines, anterior and posterior portions of each protogastric region, branchial prominences, and along margin of branchial regions (Fig. 4A).

Rostral spines divergent from base or close to it, V-shaped, up to end of antennal peduncle for smaller specimens (<4.0 mm cw) (Fig. 3C), or beyond peduncle for larger specimens (>4.0 mm cw) (Figs 3A, 4B). Angle between rostral spine and supraorbital margin obtuse and rounded (Fig. 3Ad, Ca), being not as sharp as Chace (1966) described (Fig. 3Ba). Supraorbital margin projected forward in one protruding long spine and distinct tooth at posterior angle, intercalated tooth between supraorbital margin and postocular process (Figs 3Ae, Bb, Cb, 4Aa, Ba); flat plate-shaped postocular process in dorsal view, sometimes forward (Figs 3Bc, Cc, 4Ab, Bb). Strong spine usually pointed forward, on hepatic region behind postocular process (Fig. 3Af).

Lateral margin of branchial region with four distinct spines, first two closes together, last upward and largest, nearly as large as hepatic spine (Figs 3Ag, 4H). Specimens (>4.0 mm cw) maybe exhibiting five spines, one additional smaller spine between others, as in male holotype (Fig. 4G), or four small spines arranged more regularly with last spine larger in size (Fig. 3D).

Basal antennal article with long anterior spine visible from above (between rostral spines and supraorbital margin) approaching preocular spine in length (Fig. 3Ea) and distinct tooth just posterior to posterolateral angle of basal antennal article (Fig. 3Eb). Aforementioned long spine usually with small, more or less developed distal tooth on outer edge in males and females of all sizes (Fig. 4C, F), spine only absent in two female specimens < 4.0 mm cw, or at least not visible.

Pterygostomian region with row of three acute teeth, posteriorly decreasing in size along small transversely arranged ridge; first and largest tooth on different plane than others; last tooth maybe scarcely visible (Fig. 3Ec).

Pereiopods unequal in length, longer in males than in females: P2>P3>P1 (cheliped)>P4>P5.

Cheliped: merus with prominent tubercles on dorsal midline, with large tubercle on dorsal articulation with carpus; propodus with well-defined ventral margin (resembling keeled structure). Chelipeds of male as long as carapace; merus covered with tubercles and four spines on dorsal midline; carpus with four rows of large tubercles delimiting three broad, longitudinal sulci; propodus with massive tubercle at dorsal articulation with carpus and double row of irregular tubercles or sharp granules forming shallow longitudinal sulcus on dorsal margin, ventral margin with blunt carina on proximal 3/4, outer surface microscopically roughened but not tuberculate; inner surface swollen and bearing about five pearly tubercles scattered on swollen portion; dactylus meeting in distal half, gaping proximally, with large, rectangular, serrate tooth on basal third. Chelipeds of females smaller than those of males; merus and carpus strongly spinose; chela smoother, without basal tooth on movable finger.

Walking legs decreasing in length posteriorly; merus with rounded dorsal protuberance terminally; carpus with single tubercle near meral articulation, followed by deep longitudinal furrow; propodus of P2 swollen distally in male only; dactylus with 5–7 low tubercles on ventral margin.

Male pleon and gonopods as figured by Chace (1966: fig. 14g, h).

##### Colour.

The collected material was preserved in ethanol, and its coloration had faded, unlike the specimens described by Chace (1966: 653), which, although also fixed in ethanol, still retained some of their original colour, that he described as: “carapace with rusty to orange-red reticulated mottling on grayish background; elevations cream colored. Chelipeds with dark, rusty red patch near distal end of upper surface of merus and reticulations of same color on carpus and on inner and outer surfaces of hand; light orange-red mottling on upper surface of hands and in two bands on fingers. Speckels of red on ambulatory legs and on ventral surfaces of crab.” Colour photographs, however, are available in Brown (2014: 48, 2 colour plates). She described the coloration as pale to reddish-brown, which closely corresponds to the description by Chace (1966), particularly on the legs.

##### Distribution.

Only known from its type locality, off Rupert’s Bay, Saint Helena Island (Chace 1966; present data).

##### Habitat.

Holotype (USNM 112459) and paratype (USNM 112460) were collected from a buoy and cable of a buoy, respectively, at 0–2 m depth (Chace 1966). The present specimens were collected from intertidal algal mats and a zoanthid colony, 5.2 m depth.

**Table 1.** Comparative morphological and meristic data of *Afropisasanctaehelenae* (Chace, 1966) studied in the present work.

	NHMUK 2024.232	NHMUK 2024.233	NHMUK 2024.235	NHMUK 2024.236	NHMUK 2024.237	NHMUK 2024.238	NHMUK 2024.239	NHMUK 2024.240
**Sex**	♂	ovig. ♀	ovig. ♀	♂	ovig. ♀	ovig. ♀	ovig. ♀	♂
**pcl/cw**	1.18	1.19	1.21	1.32	1.34	1.10	1.15	1.15
**rostral spines**	beyond antennal peduncle	up to antennalpeduncle	up to antennal peduncle	up to antennal peduncle	up to antennal peduncle	beyond antennal peduncle	beyond antennal peduncle	beyond antennal peduncle
**supraorbital spine (anterior)**	acute	sharp but not pointed	acute	acute	acute	acute	acute	acute
**supraorbital spine (posterior)**	acute, forward	blunt	blunt, forward	acute, forward	acute, forward	acute, forward	blunt, forward	blunt
**hepatic spine**	acute, forward	blunt	blunt, forward	acute, forward	acute, forward	acute, forward	acute, forward	blunt, forward
**lateral spines**	4 (small, not regular) + 1 (acute and forward)	4 (small, not regular) + 1 (acute and forward)	3 (small) + 1 (big) all smooth	3 (small) + 1 (acute and forward)	3 (small) + 1 (acute and forward)	4 (small and regular) + 1 (acute and forward)	3 (small) + 1 (acute and forward) + 1 (small, next to base of larger one)	3 (small)+1 (acute and forward)
**anterior spine in basal antennal article**	with small spine on outer side	with small spine on outer side	with small spine on outer side	with small spine on outer side	not developed	with small spine on outer side	with small spine on outer side	with small spine on outer side

### ﻿Molecular study

The best-fitting nucleotide substitution model for the 16S alignment (576 bp) was Tamura 3-parameter with invariant sites and gamma-distributed rates for the variable sites (T92+G+I). In the Cox1 analysis, the final alignment consisted of 658 bp, and the best-fitting nucleotide substitution model was the general-time-reversible model with invariant sites and gamma-distributed rates for the variable sites (GTR+G+I). All sequences included in both analyses are listed in Table 2.

**Table 2.** List of specimens used on molecular analysis, including specimen code of the Institution where the DNA voucher is deposited, and accession codes of the sequences deposited in GenBank database. Sequences obtained in the present work in bold. (–) No sequence available or not used in the current analysis. ^1^ Parental female of the larval development described by Guerao et al. (2003). ^2^ Deposited at Deutsches Zentrum für Marine Biodiversitätsforschung, Wilhelmshaven, Germany. ^3^ No data about DNA voucher. ^4^ DNA voucher identified as *Pisahirticornis*.

Species	Specimen code	GenBank Accession Code 16S	GenBank Accession Code COI
*Afropisacarinimana*	MMF428227	OP326676	OP326263
*Afropisacarinimana*	IEOCD-CCLME12/891	OP326677	OP326264
*Afropisacarinimana*	IEOCD-GB08/182	OP326678	—
*Afropisacarinimana*	IEOCD-PISA/3000	OP326679	OP326265
*Afropisacarinimana*	IEOCD-PISA/3001	OP326674	—
*Afropisacarinimana*	IEOCD-PISA/3002	OP326680	OP326266
*Afropisacarinimana*	IEOCD-PISA/291	OP326675	OP326262
*Afropisasanctaehelenae*	NHMUK 2024.233	**PQ788831**	**PQ788832**
*Afropisasanctaehelenae*	NHMUK 2024.235	**PQ788866**	**PQ788867**
*Apiomithraxviolaceus*	CCDB<BRA>5054	MF490139	MF490070
*Apiomithraxbocagei*	IEOCD-GB-2968	PP118335	PP133825
*Herbstiacondyliata*	IEOCD-PISA/293	OP326683	OP326269
*Micropisaovata*	IEOCD-PISA/3039-2	OP326681	OP326267
*Micropisaovata*	IEOCD-PISA/3039-3	OP326682	OP326268
*Pisaarmata*	MT04649^2^	—	KT208450
*Pisaarmata*	IEOCD-AR15/2551	—	OP326245
*Pisaarmata*	IEOCD-AR16/2552	OP326654	—
*Pisaarmata*	IEOCD-LANGAMAU/2181	OP326655	OP326247
*Pisaarmata*	IEOCD-GE17/2133	OP326652	—
*Pisachiragra*	ICMD297/2000^1^	OP326647	OP326240
*Pisachiragra*	IEOCD-PISA/294	OP326648	OP326241
*Pisahirticornis*	ICMZ2040/2016	OP326672	OP326261
*Pisahirticornis*	ICMZ13887/2017	OP326671	OP326260
*Pisamuscosa*	ICMZ13865/2017	OP326658	—
*Pisamuscosa*	IEOCD-PISA/3005	OP326657	OP326248
*Pisamuscosa*	IEOCD-PISA/3006	—	OP326249
*Pisamuscosa*	IEOCD-PISA/3007	—	OP326250
*Pisanodipes*	MMF8838	OP326662	—
*Pisanodipes*	IEOCD-PISA/3008	OP326660	OP326252
*Pisanodipes*	IEOCD-PISA/3032	OP326663	OP326253
*Pisanodipes*	SEAMoBB_Italy_BC^3^	—	ON716103
*Pisatetraodon*	MMF23312	OP326665	—
*Pisatetraodon*	UB2011PH^4^	KC866332	—
*Pisatetraodon*	IEOCD-PISA/3010	—	OP326255
*Pisatetraodon*	IEOCD-PISA/3014	OP326667	OP326256
*Pisatetraodon*	IEOCD-PISA/3038	—	OP326259
*Samadiniagalathea*	IEOCD-MZ09/1802-1	MZ424947	MZ434792
*Samadiniapulchra*	IEOCD-MZ08/1819-2	MZ424950	MZ434795

**Figure 5.**
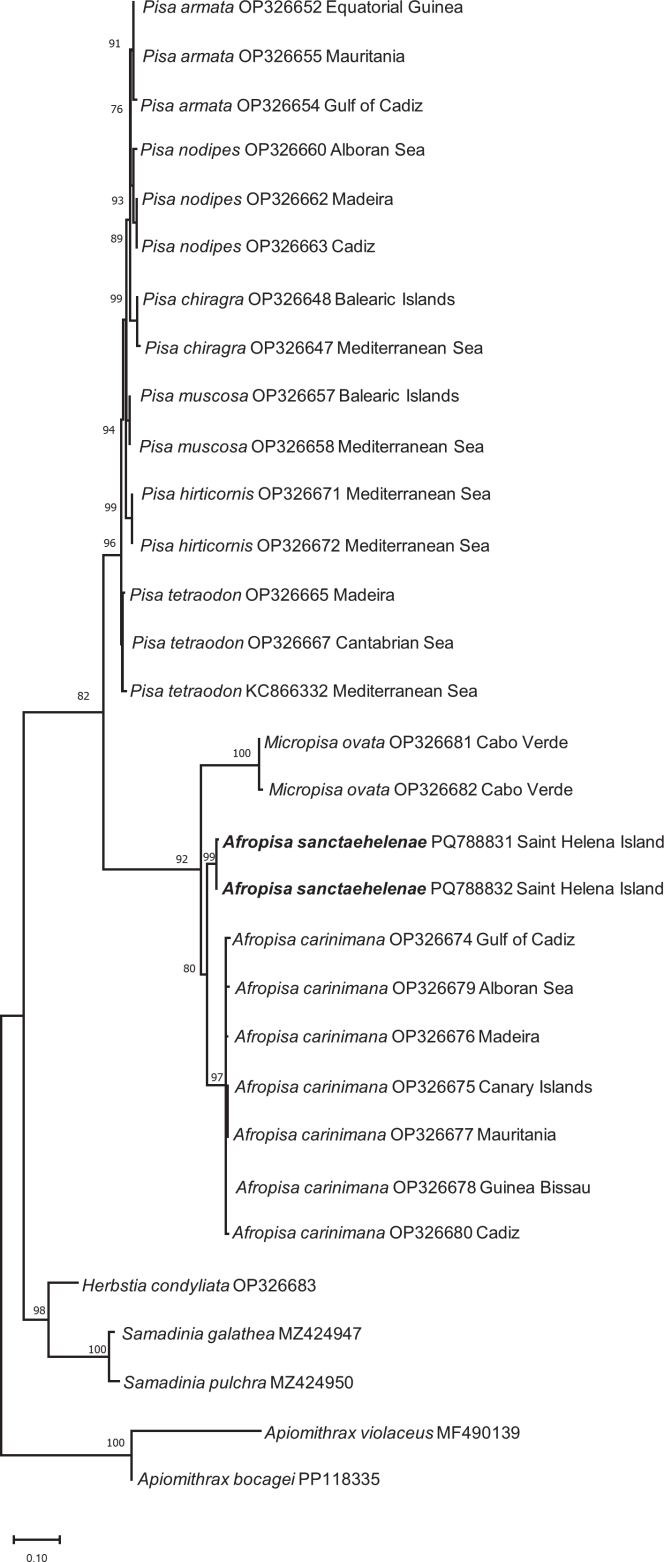
Maximum-likelihood (ML) phylogenetic tree based on mitochondrial 16S rRNA sequences of species of *Pisa*, *Afropisa*, and *Micropisa*, using 2000 bootstrap replicates. Number on nodes represent ML bootstrap values, only values > 70 are included. *Herbstiacondyliata*, *Samadiniagalathea*, *Samadiniapulchra*, *Apiomithraxviolaceus*, and *Apiomithraxbocagei* are used as outgroup. GenBank accession codes and geographic area (except for outgroup) are indicated for each species.

**Figure 6.**
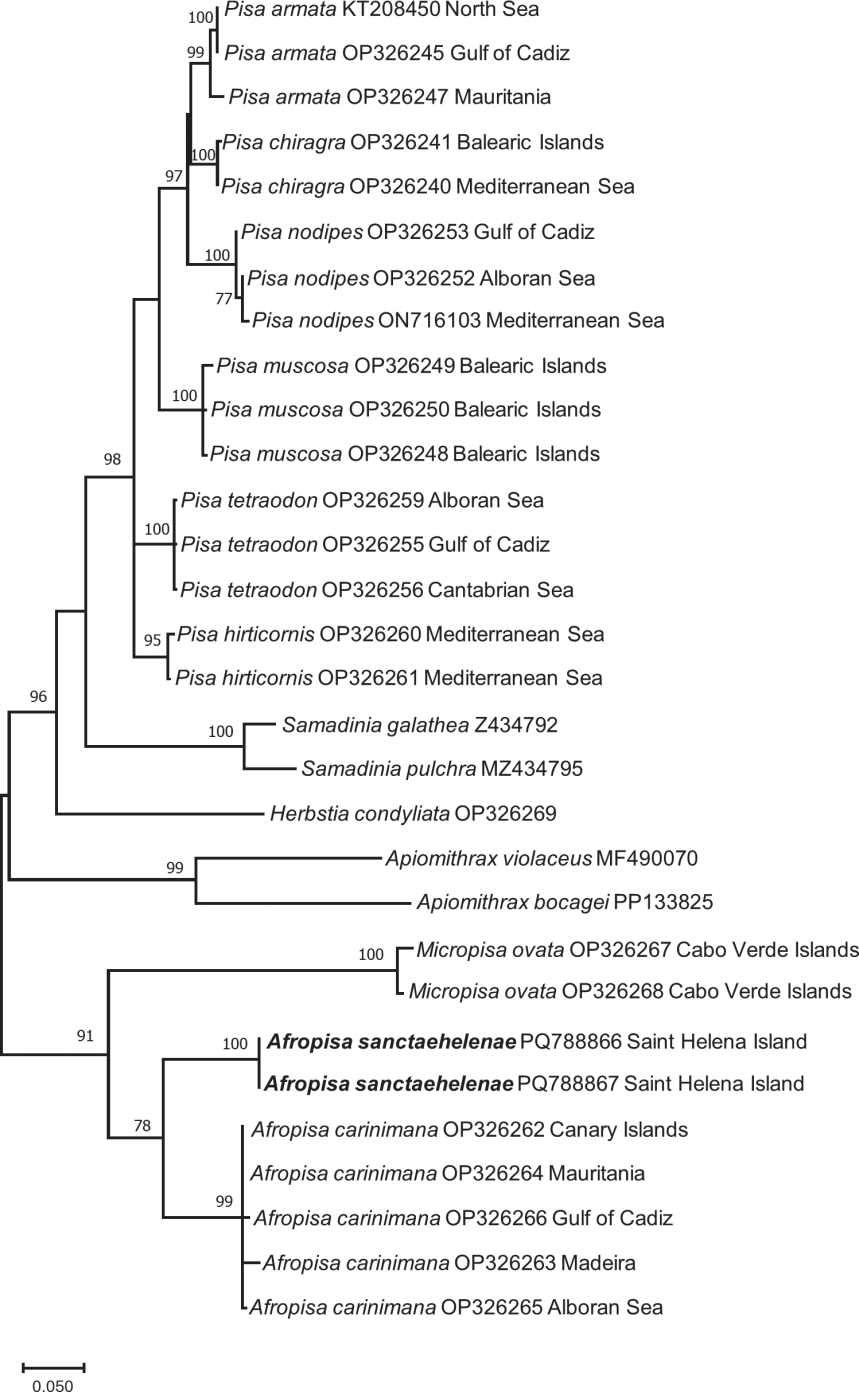
Maximum-likelihood (ML) phylogenetic tree based on mitochondrial cytochrome c oxidase subunit I sequences of species of *Pisa*, *Afropisa*, and *Micropisa*, using 2000 bootstrap replicates. Number on nodes represent ML bootstrap values, only values > 70 are included. *Herbstiacondyliata*, *Samadiniagalathea*, *Samadiniapulchra*, *Apiomithraxviolaceus*, and *Apiomithraxbocagei* are used as outgroup. GenBank accession codes and geographic area (except for outgroup) are indicated for each species.

The results of the 16S and Cox1 analyses show a separate and well-supported clade for *A.carinimana* and *A.sanctaehelenae*, which is mainly related to the clade of *M.ovata* (Figs 5, 6). The genetic distances between *A.carinimana* and *A.sanctaehelenae* are larger than commonly expected at the intrageneric level but in a similar range to those observed for species of *Samadinia* Ng & Richer de Forges, 2013, and *Apiomithrax* Rathbun, 1897, included in the current analyses of 16S and Cox1 (Figs 5, 6).

## ﻿Discussion

The generic placement of *Afropisasanctaehelenae* by Muñoz et al. (2023) was based only on morphology. The recent collections of *A.sanctaehelenae* specimens have allowed the extraction of DNA sequences and confirmed the systematic status of this species. In addition, a species redescription is possible from the newly collected specimens of *A.sanctaehelenae*, which allowed for an improvement in the original description made by Chace (1966). This redescription includes a further details of the carapace regions and row of teeth in the pterygostomian region; differences found in shape and length of rostral spines between specimen sizes (up to 4.0 mm and from 4.0 mm cw onward); variability in the number of spines on the lateral margin of the branchial regions with size (up to 4.0 mm and from 4.0 mm cw onward); a detailed description of orbital region, e.g., supraorbital margin, intercalated tooth between supraorbital margin and postocular process, and spine on hepatic region; and the presence of a spine on the basal antennule article not observed in *A.calva* or *A.carinimana.* Also, the differences in pereiopod lengths between sexes and the new relationships pcl/cw are reported herein (Table 1).

Regarding size and morphology, all eight newly collected specimens were smaller than the male holotype and two ovigerous female paratypes described by Chace (1966). This has allowed for the identification of a morphological feature that varies with size, such as the case of the rostral spines. In addition, the distal minute spine on the spine of the basal antennal article is a character not described by Chace (1966), but it is present in both the holotype, although not well developed (Fig. 4C), and the paratype (Fig. 4D).

All recent females found, even the smallest (2.9 mm cw), were ovigerous, therefore, they are adults. This agrees with one of the *Afropisa* characters in that all three species are small-sized crabs (Muñoz et al. 2023).

The sequences of 16S and Cox1 genes and the phylogenetic analysis confirm the placement of *A.sanctaehelenae* in *Afropisa* recently described by Muñoz et al. (2023). In both cases, 16S and Cox1 sequences, the distances between *A.sanctaehelenae* and *A.carinimana* are greater than expected at the intrageneric level, but similar or even higher values (see *Apiomithrax* included in the current analysis) have been found in other epialtids and majoids (Hultgren and Stachowicz 2008; Colavite et al. 2019). These genetic distances can be explained by the long-time isolation of island populations from the continent’s congeneric species (Schubart 2011). Until now, this species has only been reported from Saint Helena Island and not recorded from the closest neighbour, Ascension Island (Manning and Chace 1990). Currently, *Afropisasanctaehelenae* is considered an endemic of Saint Helena.

## Supplementary Material

XML Treatment for Afropisa sanctaehelenae

## ﻿Additional information

### Author ORCIDs

Isabel Muñoz https://orcid.org/0000-0003-1055-0754

Paul F. Clark https://orcid.org/0000-0001-6862-3982

Jose A. Cuesta https://orcid.org/0000-0001-9482-2336

### Data availability

All of the data that support the findings of this study are available in the main.
